# Diagnosis of Soil-Transmitted Helminths in the Era of Preventive Chemotherapy: Effect of Multiple Stool Sampling and Use of Different Diagnostic Techniques

**DOI:** 10.1371/journal.pntd.0000331

**Published:** 2008-11-04

**Authors:** Stefanie Knopp, Ali F. Mgeni, I. Simba Khamis, Peter Steinmann, J. Russell Stothard, David Rollinson, Hanspeter Marti, Jürg Utzinger

**Affiliations:** 1 Department of Public Health and Epidemiology, Swiss Tropical Institute, Basel, Switzerland; 2 Helminth Control Laboratory Unguja, Ministry of Health and Social Welfare, Zanzibar, Tanzania; 3 National Institute of Parasitic Diseases, Chinese Center for Disease Control and Prevention, Shanghai, People's Republic of China; 4 Wolfson Wellcome Biomedical Laboratories, Department of Zoology, Natural History Museum, London, United Kingdom; 5 Department of Medical and Diagnostic Services, Swiss Tropical Institute, Basel, Switzerland; World Health Organization, Switzerland

## Abstract

**Background:**

Soil-transmitted helminth infections are common throughout the tropics and subtropics and they disproportionately affect the poorest of the poor. In view of a growing global commitment to control soil-transmitted helminthiasis, there is a need to elucidate the effect of repeated stool sampling and the use of different diagnostic methods in areas targeted for preventive chemotherapy that are characterized by low-infection intensities. In this study, we focused on schoolchildren on Unguja Island, Zanzibar, an area where anthelminthic drugs have been repeatedly administered over the past decade.

**Methodology/Principal Findings:**

Three serial stool samples from each of 342 schoolchildren were examined using the Kato-Katz (K-K), Koga agar plate (KAP), and Baermann (BM) techniques. These methods were used individually or in combination for the diagnosis of *Ascaris lumbricoides* (K-K), *Trichuris trichiura* (K-K), hookworm (K-K and KAP), and *Strongyloides stercoralis* (KAP and BM). The examination of multiple stool samples instead of a single one resulted in an increase of the observed prevalence; e.g., an increase of 161% for hookworm using the K-K method. The diagnostic sensitivity of single stool sampling ranged between 20.7% for BM to detect *S. stercoralis* and 84.2% for K-K to diagnose *A. lumbricoides*. Highest sensitivities were observed when different diagnostic approaches were combined. The observed prevalences for *T. trichiura*, hookworm, *A. lumbricoides*, and *S. stercoralis* were 47.9%, 22.5%, 16.5%, and 10.8% after examining 3 stool samples. These values are close to the ‘true’ prevalences predicted by a mathematical model.

**Conclusion/Significance:**

Rigorous epidemiologic surveillance of soil-transmitted helminthiasis in the era of preventive chemotherapy is facilitated by multiple stool sampling bolstered by different diagnostic techniques.

## Author Summary

Diseases caused by parasitic worms inflict an enormous public health burden in developing countries. There is a growing effort to control worms with drugs. The success of repeated drug administrations can be assessed by measuring the decline in the prevalence and intensity of worm infections. Accurate diagnosis is a challenge, especially in areas with low infection intensities. We studied the effect of stool sampling efforts and the use of different diagnostic techniques on the measured prevalence of worms, including hookworm, large intestinal roundworm (*Ascaris lumbricoides*), whipworm (*Trichuris trichiura*), and dwarf threadworm (*Strongyloides stercoralis*) in Zanzibar, where worm control has been implemented over the past decade. Three early morning stool samples were collected from each of 342 schoolchildren on 3 consecutive days and analyzed with different techniques. The observed prevalence of the different worms increased with an enhanced sampling effort and when different diagnostic methods were combined. Examination of 3 stool samples per individual resulted in prevalences of *T. trichiura*, hookworm, *A. lumbricoides*, and *S. stercoralis* of 47.9%, 22.5%, 16.5%, and 10.8%, respectively. To conclude, the examination of multiple stool samples and the use of different techniques are recommended for accurate diagnosis of worms in areas undergoing repeated mass drug administration.

## Introduction

Soil-transmitted helminth infections inflict a significant burden on the world's poorest populations living in rural or deprived urban settings in developing countries [1],[2]. The most prevalent soil-transmitted helminths are *Ascaris lumbricoides*, *Trichuris trichiura* and the hookworms (*Ancylostoma duodenale* and *Necator americanus*), each parasitizing hundreds of millions of people [1]–[4]. Pre-school as well as school-aged children and pregnant women are the groups at highest risk of morbidity due to these infections [5],[6]. *Strongyloides stercoralis* is another important human helminth species, with disseminated infections being potentially fatal [7],[8].

Significant progress has been made in the control of soil-transmitted helminthiasis by means of large-scale administration of anthelminthic drugs targeting high-risk groups or entire populations. A number of initiatives to reduce helminth-related morbidity are currently underway in different countries [9]. Single-dose anthelminthic treatment, usually without prior diagnosis administered to high-risk groups, is the strategy of choice. This approach has been termed ‘preventive chemotherapy’ [10]. It is important to note, however, that cure is often not complete and depends on the anthelminthic drug utilized [11]. The predominance of light infections following anthelminthic drug administration is deemed acceptable because worm load has been convincingly linked with morbidity [1]. Additionally, a decreased number of worms results in a decline of egg excretion and, hence, in reduced environmental contamination and transmission. For both reasons the success of mass drug administration is more accurately measured if infection intensities rather than prevalences are observed [12]. The most widely used approach to assess the prevalence and infection intensity of the major soil-transmitted helminths (i.e., *A. lumbricoides*, the hookworms and *T. trichiura*) is the Kato-Katz (K-K) technique [13], which is also recommended by the World Health Organization (WHO) [14]. However, the K-K method lacks sensitivity if only a single stool sample is examined, particularly in areas with high proportions of light-intensity infections [15]. A small number of helminth eggs, unequally excreted over days and patchily distributed in stool, can occasionally not be detected in the small amount of stool examined with the K-K (i.e., 41.7 mg), hence negatively impacting on the method's sensitivity. For the detection of *S. stercoralis*, other and more labor-, material- and infrastructure-demanding methods than the K-K technique are required, turning *S. stercoralis* into a particularly neglected helminth [16]. *S. stercoralis* larvae hatch in the intestines of humans, and infections are most sensitively identified with the Koga agar plate (KAP) method [17] and the Baermann (BM) technique [18]. However, the true sensitivity of different diagnostic approaches used to detect *S. stercoralis* infections is still debated [16],[19],[20].

The aim of this study was to investigate the performance of the K-K, KAP and BM techniques, as well as combinations thereof, for the diagnosis of soil-transmitted helminth infections in an area exposed to intensive helminth control activities. The study focused on schoolchildren in two settings of Zanzibar, an island where helminth control programs, emphasizing chemotherapy-based morbidity control, have been carried out since the mid-1990s [21]–[23]. In a cross-sectional survey multiple stool samples were collected and examined with the above-mentioned methods to assess the effect of sampling effort and the use of multiple techniques for helminth-specific diagnosis.

## Materials and Methods

### Study area and population

The study was carried out on Zanzibar Island (Unguja), Tanzania, in June and July 2007. The average annual temperature in Unguja is 26.5°C. There is a long rainy season lasting from mid-March to mid-June, and a wet period with short rains from October to December.

Stool samples were obtained from children attending the primary schools of Kinyasini and Chaani, where a number of previous surveys revealed a high prevalence and infection intensity of soil-transmitted helminths [24]–[26]. Both schools are located in the district “North A”, ∼35 km northeast from Zanzibar Town, and are served by the national helminth control program which has been administering anthelminthic drugs on a fairly regular basis to school-aged children since the mid-1990s using single-dose mebendazole (500 mg) or albendazole (400 mg). For this survey, 30–50 schoolchildren from each of the 7 standards (grades) were randomly selected and invited to participate.

### Field and laboratory procedures

Participating schoolchildren were asked to submit 3 stool specimens over consecutive days. Specimens were collected in the early morning. Within 3 hours, the specimens were transported to the Helminth Control Laboratory Unguja (HCLU) located in Mianzini, Zanzibar Town, where diagnosis was initiated immediately. Specimens were processed and examined by experienced laboratory technicians from HCLU. Each specimen was investigated according to the following priorities. First, for the detection of helminth eggs, a single K-K thick smear [13] was prepared on microscope slides using the standard template holding 41.7 mg feces. After a clearing time of 40–60 min, the slides were examined under a light microscope. The number of helminth eggs was counted on a per-species basis, and recorded. For quality control, a random sample of 5% of all slides was re-examined by a senior laboratory technician.

Second, for the detection of *S. stercoralis* and hookworm larvae, the KAP procedure was performed [17]. For this purpose, agar plates were freshly prepared every evening, and stored at 4°C. A groundnut-sized portion of a stool sample (∼2 g) was placed in the middle of the agar plate. The closed Petri dish was incubated in a humid chamber for 2 days at ambient temperature. Following incubation, the plates were examined for the presence of *S. stercoralis* and hookworm larvae under a light microscope. The larvae of hookworm are usually more inert and tend to stay close to where the stool sample has been placed on the agar, whereas *S. stercoralis* larvae are more active and mobile. However, discriminative characteristics can only be determined under a microscope. Hence, the plates were rinsed with 10 ml of a 10% acetyl-formalin solution. The eluent was centrifuged at 500 g for 1 min, and the sediment was microscopically examined at 400× magnification. Hookworm and *S. stercoralis* were determined on the basis of established morphologic characteristics, i.e., the long buccal cavity and small genital primordium of hookworm larvae, and the short buccal cavity and large genital primordium of rhabditiform (L_1_) *S. stercoralis* larvae. The filariform larvae (L_3_) of the latter nematode can be identified by their characteristically forked tail.

Third, the BM technique [18] was used for *S. stercoralis* detection. For this purpose, a walnut-sized stool sample (∼10 g) was placed on a gauze inserted into a glass funnel, and covered with tap water. The apparatus was then exposed to artificial light directed to the bottom of the funnel. After 2 hours, the bottom 50 ml of the liquid was collected in a plastic tube, and spun at 500 g for 2 min. The supernatant was removed using a water suction pump. The sediment was transferred to a microscope slide, and examined under a microscope at a 100× magnification to detect, and a 400× magnification to confirm the identity of *S. stercoralis* L_1_ larvae.

### Data management and statistical analysis

All data were entered twice in Microsoft Excel version 10.0 (2002 Microsoft Corporation). Datasets were compared using EpiData version 3.1 (EpiData Association; Odense, Denmark), and discrepancies removed based on the original records.

Statistical analyses were carried out with JMP version 5.0.1 (SAS Institute; Cary, NC, United States of America). Only schoolchildren who had 3 stool samples examined with the same method or combination of methods were included in the final analyses. The number of eggs counted in the K-K thick smear was multiplied by a factor of 24 to obtain a standard infection intensity measure, which is expressed in eggs per gram of stool (EPG). The arithmetic mean EPG for each individual was calculated to summarize the EPG of stool samples submitted by the same individual. The arithmetic means were used to stratify the *A. lumbricoides*, hookworm and *T. trichiura* infection intensities according to guidelines put forward by WHO [14]. The thresholds for moderate and heavy infections were 5000 and 50,000 EPG for *A. lumbricoides*, 1000 and 10,000 EPG for *T. trichiura*, and 2000 and 4000 EPG for hookworm, respectively.

The geometric mean EPG for the whole study population was calculated taking into account both positive and negative readings of the K-K using the 10th logarithm of the EPG augmented by 1 (log(n+1)).

The sensitivity (i.e., proportion of true positives identified as positive) and negative predictive value (i.e., proportion of healthy people among negative test results) of the individual diagnostic tools and of appropriate combinations were assessed. Species-specific ‘true’ prevalences and the number of stool samples were also estimated to attain a given percentage of false negatives using the mathematical model developed by Marti and Koella [27]. This model employs the frequency of positive test results among stool samples submitted by the same individual to predict the sensitivity of the diagnostic test and to calculate the number of stool samples needed for the test to be below a given percentage of false negative results. The procedure follows an approach developed by Mullen and Prost [28], and has been employed before to predict the ‘true’ prevalences of soil-transmitted helminths, including *S. stercoralis* [16],[29],[30].

### Ethical considerations and treatment

This study was embedded in a school-based parasitological survey in Unguja, which is regularly conducted by the HCLU. Approval for the study was given by the institutional research commission of the Swiss Tropical Institute (Basel, Switzerland) and the National Health Service Local Research Ethics Committee (application 03.36) of St. Mary's Hospital (London, United Kingdom) on behalf of the Natural History Museum/Imperial College London. The study protocol was cleared by the WHO (Geneva, Switzerland), the Ministry of Health and Social Welfare (Stone Town, Zanzibar) and the Ministry of Education of Unguja (Stone Town, Zanzibar).

The headmasters of the Kinyasini and Chaani primary schools were informed about the purpose and procedures of the study. The schoolchildren were then informed by the teachers. Parents or legal guardians had given written informed consent to all anticipated medical interventions including parasitological surveys at school level when registering their child for school attendance. The children were treated regardless of their infection status with a single-dose albendazole (400 mg) within the framework of the annual mass drug administration conducted by the HCLU. Children with confirmed *S. stercoralis* infections were treated with ivermectin (single-dose, 200 µg/kg).

## Results

### Study cohort

**Figure 1:**
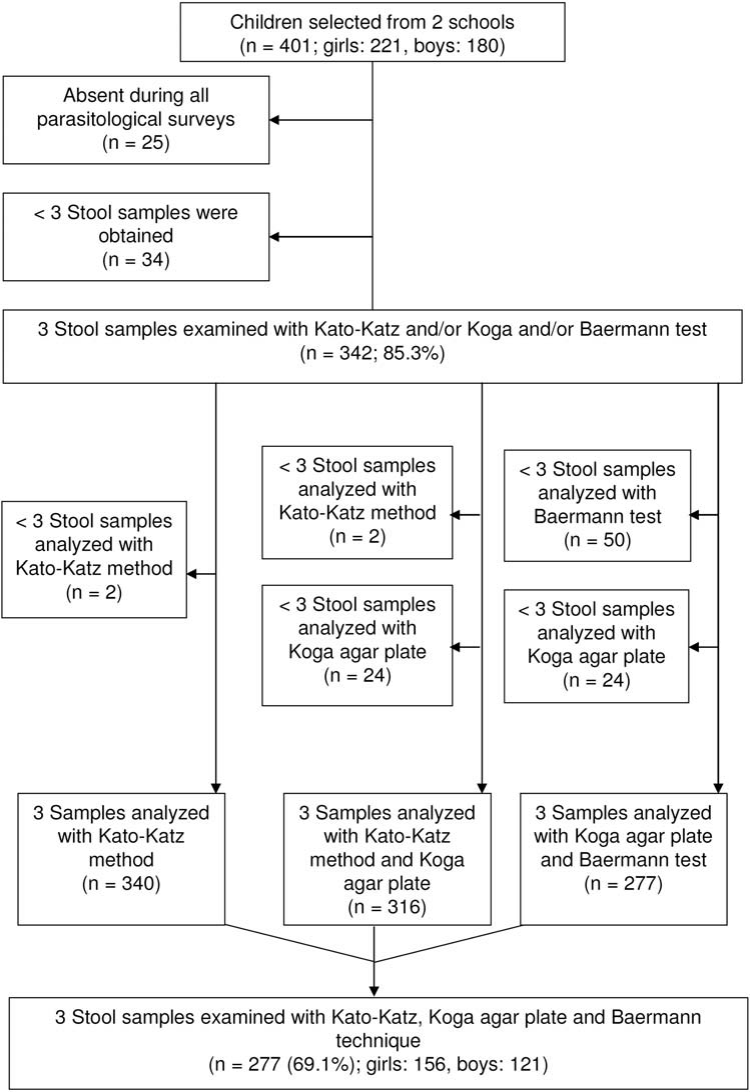
Flow chart detailing the study participation and compliance of randomly selected children from Chaani and Kinyasini schools, Zanzibar. Those children who provided 3 stool samples were included in the final analysis. The final cohort comprised those children who had complete data records, i.e., 3 stool samples examined with 3 different diagnostic methods.

Figure 1 shows that among the 401 children selected for the study in Kinyasini and Chaani schools, 221 (55.1%) were girls and 180 (44.9%) were boys. The children were aged between 7 and 20 years. The median age was 12 years and 80% of the children were between 9 and 14 years. Overall, 342 children submitted 3 stool samples over consecutive days, resulting in a compliance rate of 85.3%. Among them, 340 individuals (99.4%) had 3 samples examined with the K-K method, 318 (93.0%) with the KAP method, and 292 (85.4%) with the BM method. Since the combination of the KAP and K-K techniques (for hookworm detection) and the combination of the KAP and the BM methods (for *S. stercoralis* detection) were of particular interest, the analysis focused on 316 (97.5%) and 277 (81.0%) schoolchildren, respectively. Complete data records, i.e., 3 stool samples examined with all 3 diagnostic tests, were available for 277 out of 401 individuals, resulting in an overall compliance rate of 69.1%.

### Parasitological findings, stratified by diagnostic method

**Figure 2:**
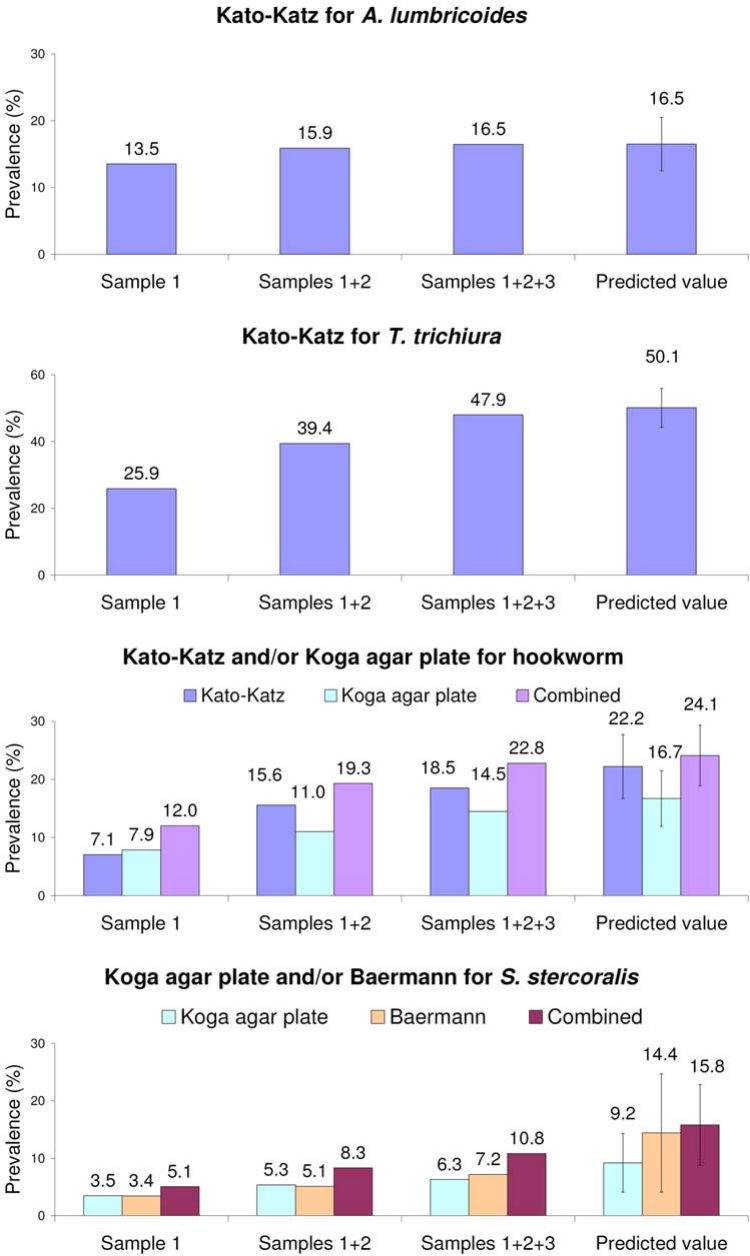
Diagrams detailing the differences in the observed and estimated ‘true’ prevalence of soil-transmitted helminth infections employing different diagnostic methods in relation to the number of stool samples from children from Chaani and Kinyasini schools, Zanzibar.

The observed intestinal helminth prevalences according to different techniques and combinations thereof, and in relation to the number of stool samples examined, are summarized in Figure 2. While the examination of 3 rather than a single stool sample by the K-K method lead to a rather small increase in the number of individuals considered *A. lumbricoides*-positive (from 13.5% to 16.5%; +22%), large increases were observed for *T. trichiura* (from 25.9% to 47.9%; +85%) and, most conspicuously, for hookworm (from 7.1% to 18.5%; +161%).

A similar trend was observed for hookworm and *S. stercoralis* when results from the KAP tests were considered. The examination of 3 instead of a single stool sample by the KAP method resulted in increases of the observed prevalences of both hookworm and *S. stercoralis* of more than 80% (from 7.9% to 14.5% for hookworm, and from 3.5% to 6.3% for *S. stercoralis*). Regarding the BM method, the analysis of 3 stool samples raised the prevalence of *S. stercoralis* from 3.4% (first sample) to 7.2% (all 3 samples; +112%).

The occurrence of hookworm and *S. stercoralis* was investigated by a combination of 2 different methods. The use of the KAP method increased the number of hookworm infections diagnosed by the K-K technique by a factor of 1.2, detecting an additional 11 infections. For *S. stercoralis*, the number of the BM-positives was surpassed by a factor of 1.5 or an additional 10 infections if the KAP results were also considered.

Using the data of 3 stool specimens, analyzed with each method or combination of methods, and Marti and Koella's mathematical model [27] revealed ‘true’ prevalences. Prevalences were calculated of 50.1% (standard deviation (SD) = 5.8%) for *T. trichiura*, and 16.5% (SD = 4.0%) for *A. lumbricoides* based on 3 K-K thick smears; 24.2% (SD = 5.2%) for hookworm using 3 K-K plus 3 KAP; and 15.8% (SD = 7.0%) for *S. stercoralis* employing 3 KAP plus 3 BM tests.

### Infection characteristics

The population geometric mean EPG values for *A. lumbricoides*, hookworm and *T. trichiura* obtained with the K-K method are summarized in Table 1, together with the maximum EPG found in a single stool sample and the infection intensities among the positives, stratified by common intensity classes. According to the WHO-defined infection intensity classification [14], most infected schoolchildren included in the final analyses had low-intensity infections; 98.4% for hookworm (1–1999 EPG), 98.1% for *T. trichiura* (1–999 EPG) and 73.2% for *A. lumbricoides* (1–4999 EPG). No high-intensity infections were found for any of the soil-transmitted helminths. Children with moderate infection intensity of *A. lumbricoides* (5000–49,999 EPG) *T. trichiura* (1000–9999 EPG) and hookworm (2000–3999 EPG) were consistently diagnosed positive in all 3 stool samples, whereas light-intensity infections were sometimes diagnosed in only 1 or 2 of the 3 samples.

**Table 1:** Characteristics of soil-transmitted helminth infections among children from Chaani and Kinyasini schools, Zanzibar, as determined by the Kato-Katz technique.

Parasite species	No. of children examined	No. (%) of children infected	Geometric mean (EPG)^a^	Maximum EPG count	No. of infected children stratified by infection intensity (values in brackets are percentage, %)			Method
					Light	Moderate	Heavy	
*T. trichiura*	340	162 (47.7)	0.77	2880	159 (98.1)	3 (1.9)	0 (0.0)	Kato-Katz
Hookworm	340	63 (18.5)	0.22	2400	62 (98.4)	1 (1.6)	0 (0.0)	Kato-Katz
*A. lumbricoides*	340	56 (16.5)	0.53	17,520	41 (73.2)	15 (26.8)	0 (0.0)	Kato-Katz

a EPG = eggs per gram of feces based on Kato-Katz thick smear examination.

The majority of *S. stercoralis* infections (85.0%) were diagnosed in only 1 of the 3 stool samples examined with the BM technique. Three children had 2 positive samples, but only 1 child provided 3 positive samples.

### Performance of the diagnostic methods

As shown in Table 2, the sensitivity of the diagnostic methods rose considerably when 3 stool samples were examined instead of a single one. The highest sensitivities were observed for the diagnosis of *A. lumbricoides* (99.6%) and *T. trichiura* (95.1%) when analyzing 3 stool samples with the K-K method. Also for hookworm and *S. stercoralis* detection, the employed methods showed highest sensitivities when 3 stool samples were examined. The combination of K-K plus KAP for hookworm diagnosis had a markedly higher sensitivity (93.1%) compared to either the K-K (83.3%) or the KAP method alone (86.8%). The combination of KAP plus BM for the identification of *S. stercoralis* showed an increased sensitivity compared to the BM technique (sensitivity of 68.5% *versus* 50.0%) but was equal to the sensitivity of the KAP method alone (68.5%). The negative predictive value of all employed methods and method combinations was above 92% if 3 stool samples were analyzed.

**Table 2:** Sensitivity of individual and combined diagnostic methods if 1 or 3 stool samples from children from Chaani and Kinyasini schools, Zanzibar, were examined (all values expressed as percentage, %) and samples needed to obtain ≤1% false negative test results.

	Kato-Katz method	Koga agar plate method	Baermann method	Kato-Katz plus Koga agar plate method	Koga agar plate plus Baermann method
*A. lumbricoides*					
Sensitivity of method (3 samples)	99.6	–	–	–	–
Sensitivity of individual test (SD)	84.2 (5.8)	–	–	–	–
Negative predictive value	99.9	–	–	–	–
Samples needed if ≤1% false negatives are allowed	3	–	–	–	–
*T. trichiura*					
Sensitivity of method (3 samples)	95.1	–	–	–	–
Sensitivity of individual test (SD)	63.4 (5.0)	–	–	–	–
Negative predictive value	95.3	–	–	–	–
Samples needed if ≤1% false negatives are allowed	5	–	–	–	–
Hookworm					
Sensitivity of method (3 samples)	83.3	86.8	–	93.1	–
Sensitivity of individual test (SD)	45.0 (9.3)	49.0 (10.6)	–	59.0 (7.9)	–
Negative predictive value	95.5	97.4	–	97.9	–
Samples needed if ≤1% false negatives are allowed	8	7	–	5	–
*S. stercoralis*					
Sensitivity of method (3 samples)	–	68.5	50.0	–	68.5
Sensitivity of individual test (SD)	–	32.0 (16.8)	20.7 (15.4)	–	32.0 (13.7)
Negative predictive value	–	96.9	92.3	–	94.4
Samples needed if ≤1% false negatives are allowed	–	12	20	–	12

The samples size needed, if up to 1% false negative results were considered acceptable, was 3 and 5 samples for *A. lumbricoides* and *T. trichiura* using the K-K method, 8 and 7 samples for hookworm with the K-K or KAP method, respectively, and 12 or 20 stool samples for *S. stercoralis* with the KAP or BM method, respectively. Combining the latter 2 methods, 5 and 12 stool samples were necessary for the diagnosis of hookworm and *S. stercoralis*, respectively.

## Discussion

There is a paucity of high-quality data regarding the effect of stool sampling effort and the use of different techniques for the diagnosis of soil-transmitted helminths in different epidemiologic settings. In particular, information is lacking on the performance of widely employed diagnostic tools in the current era of preventive chemotherapy [10]. Our findings were obtained from investigating fecal samples from more than 300 schoolchildren in Zanzibar. Children were screened over multiple days using 3 different fecal examination methods and our results confirm that an increased sampling effort and the use of multiple diagnostic approaches result in higher observed helminth prevalences [15],[16],[30]. For example, while the observed hookworm prevalence was only 7.1% after examination of a single K-K thick smear, the cumulative prevalence after screening 3 stool samples with the K-K method was more than twice as high (18.5%). Using both the K-K and the KAP method on 3 stool samples resulted in an observed prevalence of 22.8%. This observed prevalence is close to the modeled prevalence of 24.1%. The effect of multiple sampling on the observed prevalence is also notable for *S. stercoralis* and *T. trichiura*. It was, however, much less obvious with regard to the detection of *A. lumbricoides*.

Since the measured prevalence of individual helminth species increased considerably as a function of sampling effort, the diagnostic sensitivity of single stool samples may be insufficient. Indeed, the examination of only 1 stool sample considerably underestimated *T. trichiura*, hookworm and *S. stercoralis* infections. If 3 stool samples were examined, the sensitivity of all tests increased and the negative predictive values were consistently above 90%. The low sensitivity of single tests in the current setting can be attributed to the predominantly light infections among the pupils of Chaani and Kinyasini schools. Both schools are covered by the national helminth control program, which has distributed single-dose mebendazole (500 mg) and albendazole (400 mg) once or several times yearly since 1995 and 2003, respectively. Additionally, the repeated rounds of mass administration of ivermectin and albendazole as part of the global program to eliminate lymphatic filariasis, which has been implemented since 2001 [22],[31] and targets the whole island of Unguja, has also impacted on the worm load in children. The administration of ivermectin (200 µg/kg) through the latter program has most probably had a considerable impact on *A. lumbricoides* and *S. stercoralis*, since ivermectin exhibits significant ascaricidal and strongyloidicidal activity. Another factor lowering the sensitivity of diagnostic tests is day-to-day variation in fecal egg output [32],[33].

It is also known that the detection of hookworm eggs is influenced by delays in stool processing [34] and, when using the K-K technique, the time from slide preparation to reading under a microscope [35]. In this study, slides were examined within 40–60 min after preparation, which is the upper limit of the recommended clearing time [35], especially if the warm climate is taken into account. The time from stool production in the early morning until the fecal samples reached the bench – at least 3 hours – might also have impacted on the diagnostic sensitivity. Moreover, there was considerable variation between individuals and from one day to another. A time delay of more than 3 hours from stool production to examination reduced the sensitivity of the K-K method for hookworm diagnosis by almost 50% in a recent study carried out in Malawi [34]. The KAP method also allows hatching of hookworm larvae. Thus, the KAP technique can supplement the K-K method and, in the present study, the combination of these 2 methods yielded a sensitivity of 93.1% after examination of 3 stool samples per individual. A higher sensitivity for the KAP method was found compared to the K-K technique for the diagnosis of hookworm, which is in agreement with recent observations made in China [16]. However, the KAP procedure requires some basic laboratory infrastructures that are often not available in developing country settings, multiple days for incubation in a humid chamber and trained laboratory personnel [17].

It is conceivable that in some cases *S. stercoralis* larvae failed to leave the stool sample placed in the middle of the agar plate (KAP) or on the gauze embedded in the glass funnel (BM). This will result in false-negative diagnoses as the larvae can only be detected when moving on the surface of the agar plate or settling at the bottom of the funnel. Hence, relying on stool examination only will result in a certain number of false negatives. Moreover, *S. stercoralis* larvae can replicate within the host and autoinfection is possible without larvae being excreted, thus not all infections can be detected by parasitological techniques. It has been suggested that multiple stool sampling and the combination of several diagnostic methods reveal *S. stercoralis* infections with the highest sensitivity [36]. The observed increase in sensitivity obtained by combining the BM and the KAP methods coincides with results of de Kaminsky [19], but is in contrast with a recent study done by Steinmann and colleagues [16] where the BM technique identified ‘all’ cases. However, we recommend the concurrent use of both methods as each of them has strengths and limitations. Regarding the KAP method, it is not easy to perform under field conditions and requires expertise in differentiating *S. stercoralis* from hookworm larvae and, potentially, also from environmental nematodes. Additionally, agar plates containing infective larvae pose a biohazard and need to be handled and disposed of with care. Regarding the BM technique, it is less time consuming and detected larvae can be identified more easily. The most notable disadvantage of this method is the large quantity of stool needed, and hence compliance is an issue.

The calculated number of stool samples needed to reach a rate of ≤1% false negative diagnoses resulted in high numbers except for *A. lumbricoides*, where 3 samples subjected to the K-K method were sufficient. For accurate diagnosis of *T. trichiura*, hookworm and *S. stercoralis*, it was found that up to 20 stool samples need to be examined in this low intensity setting. This is not feasible except for specialized small-scale studies. Therefore, diagnostic methods with higher sensitivity and low technical demands are urgently needed. Sero-diagnosis of soil-transmitted helminths might be an option, but this approach has some disadvantages such as its more invasive nature (i.e., blood collection), the persistence of antibodies after treatment and potential cross-reactivity with other nematodes. The non-invasive FLOTAC technique [37],[38] holds promise to fill this gap and a broad-scale validation of this tool for species-specific helminth diagnosis is underway.

We conclude that in epidemiologic settings characterized by low-infection intensities of soil-transmitted helminths, it is important to examine multiple stool samples in order to avoid underestimating the ‘true’ prevalence of soil-transmitted helminth infections, and hence their transmission potential. Our results indicate that for rigorous epidemiologic surveillance, a combination of methods is required to more accurately assess the situation. From a more general infectious diseases perspective, our observations could potentially better pinpoint interactions between helminthiasis and other tropical diseases (e.g., malaria), which are likely modulated by chronic worm infections even of low egg/larvae output [39]. The discovery, development and deployment of new tools for the diagnosis and quantification of soil-transmitted helminth infections, including *S. stercoralis*, is of considerable importance for successful helminth control, and remains a research priority [40]–[42].

## Supporting Information

Alternative Language Abstract S1 Translation of the Abstract into German by S. Knopp. Diagnose von durch Bodenkontakt übertragenen Wurmerkrankungen in der Ära präventiver Chemotherapie: Bedeutung von wiederholten Stuhluntersuchungen und unterschiedlichen diagnostischen Methoden
(0.10 MB PDF)
