# Supplementary material for: Diagnosis of Soil-Transmitted Helminths in the Era of Preventive Chemotherapy: Effect of Multiple Stool Sampling and Use of Different Diagnostic Techniques
Source: PLoS Negl Trop Dis. 2008 Nov 4;2(11):e331. doi: 10.1371/journal.pntd.0000331 (PMC2570799; doi:10.1371/journal.pntd.0000331)
Supplement: Alternative Language Abstract S1 — Translation of the Abstract into German by S. Knopp. Diagnose von durch Bodenkontakt übertragenen Wurmerkrankungen in der Ära präventiver Chemotherapie: Bedeutung von wiederholten Stuhluntersuchungen und unterschiedlichen diagnostischen Methoden (0.10 MB PDF) [file pntd.0000331.s001.pdf]

Diagnose von durch Bodenkontakt übertragenen Wurmerkrankungen in der Ära präventiver Chemotherapie: Bedeutung von wiederholten Stuhluntersuchungen und unterschiedlichen diagnostischen Methoden

Zusammenfassung

Hintergrund: Infektionen mit durch Bodenkontakt übertragenen Würmern treten in den Tropen und Subtropen häufig auf und belasten besonders die ärmsten Bevölkerungsgruppen. In Hinsicht auf die wachsenden weltweiten Bemühungen diese Wurmerkrankungen einzudämmen muss der Nutzen von wiederholten Stuhluntersuchungen und die Anwendung von unterschiedlichen diagnostischen Methoden in Gebieten, in welchen präventive Chemotherapie angewandt wird und welche somit von niedriger Infektionsintensität gekennzeichnet sind, ermittelt werden. In dieser Studie legen wir den Schwerpunkt auf die Untersuchung von Schulkindern aus Unguja, Sansibar, einer Insel, auf welcher Antiwurm-Medikamente im letzten Jahrzehnt regelmäßig verabreicht wurden.

Methoden/wichtigste Ergebnisse: Drei Stuhlproben in Folge wurden von 342 Schulkindern eingesammelt und mit der Kato-Katz (K-K), Koga Agarplatten (KAP) und Baermann (BM) Methode untersucht. Diese Methoden wurden einzeln oder in Kombination für die Diagnose von *Ascaris lumbricoides* (K-K), *Trichuris trichiura* (K-K), Hakenwürmern (K-K und KAP) und *Strongyloides stercoralis* (KAP und BM) angewandt. Die Untersuchung von mehreren anstelle einer einzelnen Stuhlprobe führte zu einem Anstieg der registrierten Prävalenz; z.B. einem Anstieg von 161% für Hakenwurminfektionen, wenn die K-K Methode benutzt wurde. Die Sensitivität der Untersuchung einer einzelnen Stuhlprobe bewegte sich zwischen 20.7% für die BM Methode um *S. stercoralis* nachzuweisen, und 84.2% für die K-K Methode um *A. lumbricoides* zu diagnostizieren. Höhere Sensitivitäten konnten beobachtet werden wann

immer unterschiedliche diagnostische Methoden kombiniert wurden. Die Prävalenzen für *T. trichiura*, Hakenwürmer, *A. lumbricoides* and *S. stercoralis* betrugen 47.9%, 22.5%, 16.5% und 10.8% wenn 3 Stuhlproben untersucht wurden. Diese Werte liegen nahe der `wahren´ Prävalenzen, die durch ein mathematisches Model vorhergesagt werden.

Schlussfolgerung/Bedeutung: Die genaue epidemiologische Überwachung von Wurminfektionen im Zeitalter der präventiven Chemotherapie wird durch die Untersuchung von mehreren Stuhlproben mit unterschiedlichen Methoden gefördert.

Übersetzung: Stefanie Knopp
